# Volatile organic compounds orchestrating microbiome‐mediated crop resilience: Prospects and challenges for sustainable agriculture

**DOI:** 10.1111/jipb.70333

**Published:** 2026-07-10

**Authors:** Peiguo Yuan, Esau De la Vega‐Camarillo, Michael V. Kolomiets, Sanjay Antony‐Babu

**Affiliations:** ^1^ Department of Plant Pathology and Microbiology Texas A&M University College Station 77843 TX USA

## Abstract

Herbivory triggers the production of green leaf volatiles, and dense planting results in induces increased concentration of linalool emission in maize canopies. These volatiles activate plant‐soil feedback that enriches beneficial rhizosphere bacteria, enhancing resistance, though sometimes at a growth cost, thereby offering new avenues for sustainable crop improvement.

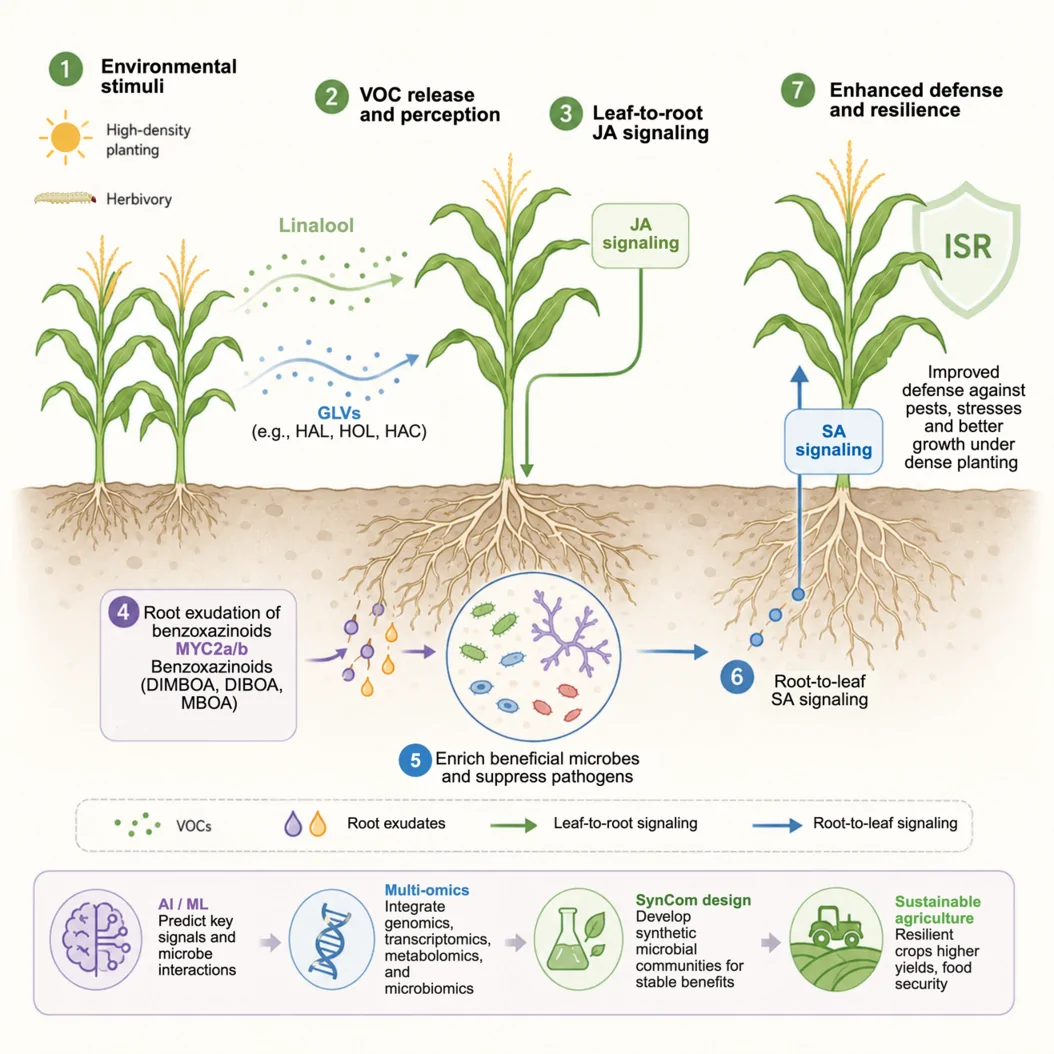

The global population is projected to reach 9.8 billion by 2050, making food security a pressing global challenge. Beyond improving individual plant yield, growing crops at higher densities while maintaining productivity and resilience is a powerful driver of agricultural output. Modified plant architecture, such as narrower statures, more upright leaves, and semi‐dwarfing, minimizes mutual shading and enhances photosynthetic efficiency under dense planting (Tian et al., 2019). However, are these structural adaptations complemented by metabolic potential that aids plant defenses?

Plant volatile signal, volatile organic compounds (VOCs), are airborne chemicals released to communicate with surrounding organisms. Mechanical wounding or herbivore attack induces VOC release at damaged sites. In addition, environmental stresses, such as extreme temperatures and drought, regulate VOC biosynthesis and membrane translocation, in part through ATP‐binding cassette (ABC) transporters. These signals alert neighboring plants to threats, thereby priming defense responses (Bouwmeester et al., 2019). Importantly, recent studies reveal that plant–soil feedback (PSF) is an essential downstream component of VOC emissions regulating plant performance. As shown in Figure 1, Hu et al. (2025) demonstrated that herbivory‐induced green leaf volatiles (GLVs), including (*Z*)‐3‐hexenal (HAL), (*Z*)‐3‐hexen‐1‐ol (HOL), and (*Z*)‐3‐hexenyl acetate (HAC), reshape the rhizosphere microbiome structure and function, which enhance both growth and defense in maize. Similarly, Guo et al. (2025) showed that high‐density planting can increase linalool accumulation that subsequently improves maize defense but represses growth through PSF. Unlike stress‐induced fatty acid‐derived volatiles such as GLVs and pentyl leaf volatiles (PLVs), and herbivore‐induced plant volatiles (HIPVs), linalool is continuously emitted at low levels, accumulating in higher concentrations under increased plant density. Thus, distinct environmental stimuli selectively activate specific volatile signals, which in turn induce unique signaling pathways through PSF to optimize plant fitness.

**Figure 1:**
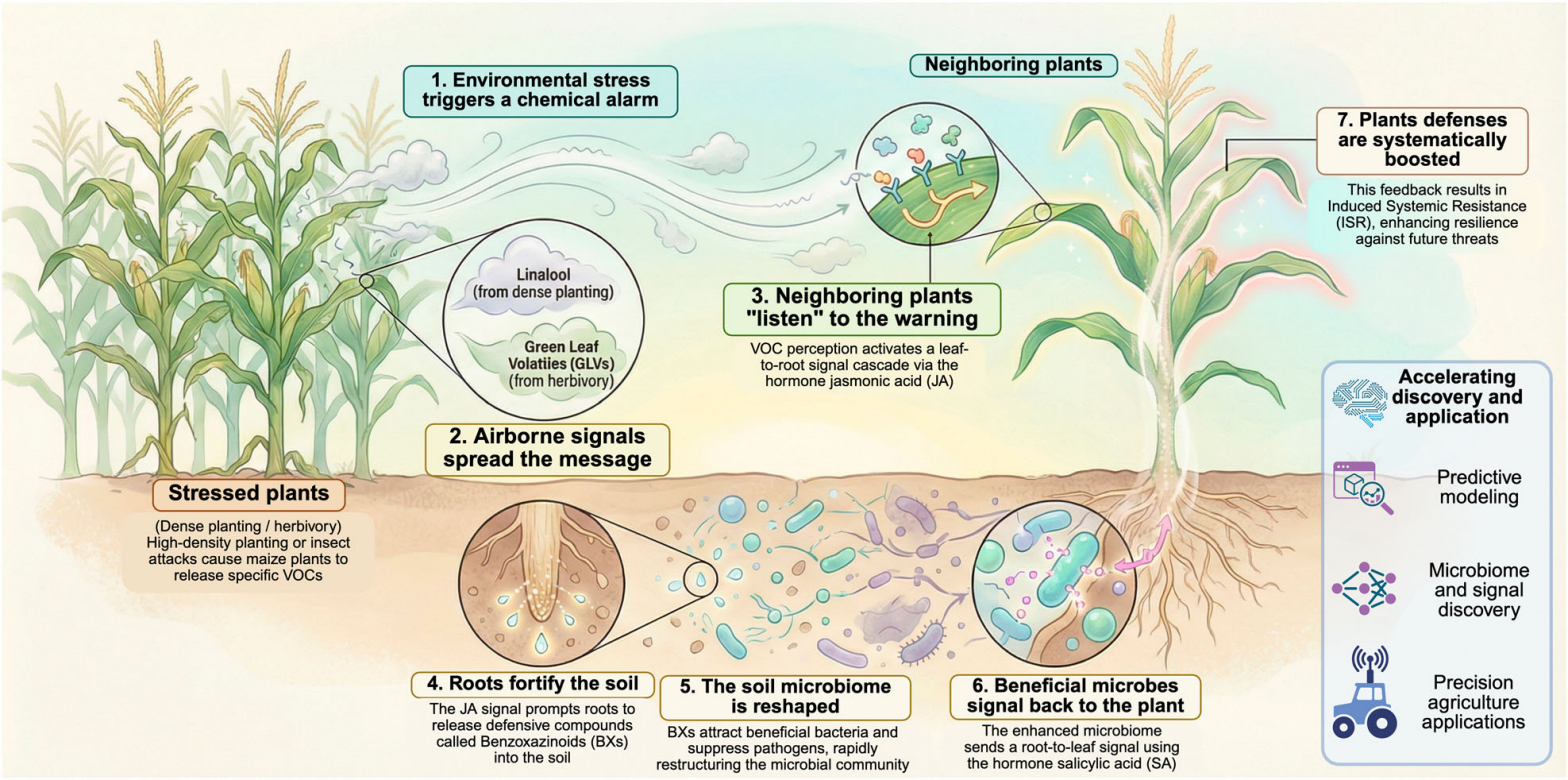
Working model illustrating how environmental stimuli trigger volatile organic compound signaling pathways and rhizosphere microbiome reshaping to enhance crop resilience 1. Environmental stimuli trigger volatile release. 2. High‐density planting enhances constitutive volatile (linalool) accumulation, while herbivory triggers damage‐induced GLVs, both initiating metabolic and ecological responses. 3. Neighboring plants perceive these volatiles to activate JA signaling from leaves to roots, modulating metabolic pathways. 4. JA signaling via MYC2a/b transcription factors induces Benzoxazinoids (BX) biosynthesis and exudation (DIMBOA, DIBOA, and MBOA) along with other natural defense compounds. 5. BX release enriches beneficial microbes and suppresses soil‐borne pathogens. 6. Rhizosphere microbiome enhances root‐to‐leaf SA signaling. 7. SA‐mediated systemic signals integrate microbial cues into ISR. Artificial intelligence (AI) can further accelerate the identification of key signaling molecules, predict plant–microbiome interactions, integrate multi‐omics data sets, and support the development of microbiome‐based solutions and precision agriculture strategies. Conceptual drafts were initially drawn by hand, enhanced via Google NotebookLM, and finalized using BioRender.

Perception of specific volatile signals in leaves activates systemic leaf‐to‐root signaling, altering root exudation profiles to selectively shape rhizosphere microbiome assembly. Guo et al. (2025) demonstrated that density‐mediated high linalool activates jasmonic acid (JA) signaling, thereby inducing benzoxazinoids (BXs) biosynthesis by engaging the MYC2a and MYC2b transcription factors. BXs play a central role in plant defense as natural insecticides and antimicrobials, while inhibiting weed growth. In maize, BXs such as 2,4‐dihydroxy‐7‐methoxy‐1,4‐benzoxazin‐3‐one (DIMBOA) and 2,4‐dihydroxy‐1,4‐benzoxazin‐3‐one (DIBOA) are synthesized in tissues and released into the rhizosphere and broken down into bioactive products such as MBOA. These compounds enrich beneficial bacteria, suppress soilborne pathogens, and modulate interactions with beneficial fungi such as mycorrhizae and *Trichoderma* (Hu et al., 2018; Kudjordjie et al., 2019; Wang et al., 2025). In short, linalool‐induced JA signaling promotes BX release, restructuring the microbial community and enhancing the defense capacity of subsequent plants grown in the same soil.

Notably, VOC‐mediated microbiome restructuring is rapid: Linalool shifts communities within 3 d; a 1‐h HAC treatment reshapes the rhizosphere microbiome within 1 d post‐treatment (Hu et al., 2025).

Root metabolite–mediated microbiome assembly activates systemic root‐to‐leaf signaling that enhances plant performance. While JA regulates leaf‐to‐root signaling for linalool‐mediated microbiome restructuring, salicylic acid (SA) modulates root‐to‐leaf systemic signaling in beneficial microbial‐induced defense against herbivory. This cascading effect has also been observed in both ryegrass and barley, suggesting a conserved mechanism across species (Guo et al., 2025). However, the specific mobile signals induced by beneficial microbes remain unclear. For example, methyl salicylate (MeSA), but not SA, acts as the mobile signal for systemic acquired resistance (SAR) (Park et al., 2007). Additionally, beneficial fungi such as *Trichoderma virens* regulate induced systemic resistance (ISR) by mediating xylem‐mobile long‐distance oxylipin signals, including the JA precursor, 12‐oxo‐phytodienoic acid (12‐OPDA), and an α‐ketol, 9,10‐ketooctadecadienoic acid (9,10‐KODA), but not JA (Wang et al., 2020).

The above studies offer novel insights that may inform strategies in the development of synthetic microbiota communities, sustainable agriculture, and climate‐smart crops. However, there are several challenges that need to be addressed before these concepts can be translated: (1) Identifying potential receptor(s) for VOC or uncovering perception mechanisms is essential for designing crops optimized for high‐density planting. Feasible strategies include genetics‐assisted receptor mining and forward genetic screens coupled with volatile treatments. (2) Expanding current bacteria‐centric studies by assessing how VOC signals influence the broader holobiome, including fungi and protists, which play critical roles in nutrient cycling, pathogen suppression, and stress resilience. Integrated microbiome pipelines combining GC‐MS‐guided microbial VOC profiling, clustering/classification approaches, and synthetic community reconstruction will be vital for testing causality. (3) Deciphering how the dosage and composition of root exudates shape microbial recruitment. Experimental frameworks using controlled soil‐conditioning gradients coupled with multi‐omics readouts will be crucial for quantifying dose‐dependent effects to engineer target root exudation patterns. (4) The impact of environmentally triggered signals on the phyllosphere microbiome remains largely unexplored. Understanding how plants regulate above‐ground communities, whether rhizosphere shifts influence phyllosphere, and how foliage microbes contribute to ISR is critical for coordination of above‐ and below‐ground microbiomes. (5) The rapid growth of meta‐omics approaches generates vast, complex data sets on plant–microbiome interactions. Artificial intelligence (AI) and machine learning (ML), particularly deep learning, offer powerful tools to handle these high‐dimensional data, model volatile signaling pathways, and predict microbiome restructuring (Hernández Medina et al., 2022). (6) Translating these concepts requires balancing short‐term productivity gains with long‐term soil health and ecosystem function. A critical challenge lies in designing synthetic microbial communities (SynComs) with high colonization efficiency and environmental compatibility—ensuring that introduced beneficial microbes can establish, persist, and function across diverse soil types and climatic conditions. Furthermore, crop diversification strategies, including intercropping, cover cropping, and rotation systems, may amplify positive feedbacks by maintaining continuous volatile signaling and sustaining beneficial microbial populations across growing seasons. Agricultural management practices that integrate volatile‐optimized crop genotypes with precision soil conditioning could reinforce these feedbacks while preserving long‐term soil fertility. We hypothesize that pairing VOC‐responsive crop genotypes with tailored SynComs within diversified cropping systems will exert synergistic effects on pathogen suppression and yield stability. The testable prediction offers a potential framework to guide next‐generation breeding programs and microbiome engineering efforts at enhancing crop resilience, sustainable agriculture, and global food security.

## CONFLICTS OF INTEREST

The authors declare no conflicts of interest.

## AUTHOR CONTRIBUTIONS

S.A. conceptualized the manuscript. P.Y., E.D.L.V., M.V.K., and S.A. wrote and revised the manuscript. All authors have read and approved the final version of the manuscript.
